# The Impact of Anxiety-Inducing Distraction on Cognitive Performance: A Combined Brain Imaging and Personality Investigation

**DOI:** 10.1371/journal.pone.0014150

**Published:** 2010-11-30

**Authors:** Ekaterina Denkova, Gloria Wong, Sanda Dolcos, Keen Sung, Lihong Wang, Nicholas Coupland, Florin Dolcos

**Affiliations:** 1 Department of Psychiatry, University of Alberta, Edmonton, Canada; 2 Centre for Neuroscience, University of Alberta, Edmonton, Canada; 3 Department of Psychology, University of Illinois, Urbana-Champaign, Illinois, United States of America; 4 Brain Imaging and Analysis Center, Duke University, Durham, North Carolina, United States of America; 5 Beckman Institute, University of Illinois, Urbana-Champaign, Illinois, United States of America; University of Barcelona, Spain

## Abstract

**Background:**

Previous investigations revealed that the impact of task-irrelevant emotional distraction on ongoing goal-oriented cognitive processing is linked to opposite patterns of activation in emotional and perceptual vs. cognitive control/executive brain regions. However, little is known about the role of individual variations in these responses. The present study investigated the effect of trait anxiety on the neural responses mediating the impact of transient anxiety-inducing task-irrelevant distraction on cognitive performance, and on the neural correlates of coping with such distraction. We investigated whether activity in the brain regions sensitive to emotional distraction would show dissociable patterns of co-variation with measures indexing individual variations in trait anxiety and cognitive performance.

**Methodology/Principal Findings:**

Event-related fMRI data, recorded while healthy female participants performed a delayed-response working memory (WM) task with distraction, were investigated in conjunction with behavioural measures that assessed individual variations in both trait anxiety and WM performance. Consistent with increased sensitivity to emotional cues in high anxiety, specific perceptual areas (fusiform gyrus - FG) exhibited *in*creased activity that was positively correlated with trait anxiety and negatively correlated with WM performance, whereas specific executive regions (right lateral prefrontal cortex - PFC) exhibited *de*creased activity that was negatively correlated with trait anxiety. The study also identified a role of the medial and left lateral PFC in coping with distraction, as opposed to reflecting a detrimental impact of emotional distraction.

**Conclusions:**

These findings provide initial evidence concerning the neural mechanisms sensitive to individual variations in trait anxiety and WM performance, which dissociate the detrimental impact of emotion distraction and the engagement of mechanisms to cope with distracting emotions. Our study sheds light on the neural correlates of emotion-cognition interactions in normal behaviour, which has implications for understanding factors that may influence susceptibility to affective disorders, in general, and to anxiety disorders, in particular.

## Introduction

It is generally accepted that susceptibility to mood and anxiety disorders is linked to individual differences in the processing of emotional information. However, the role of personality-related differences affecting emotion processing and the associated neural correlates are not clear. Here, we used functional magnetic resonance imaging (fMRI) in conjunction with behavioural and personality measures to investigate the effects of anxiety-related individual differences on the neural circuitry underlying the response to transient anxiety-inducing emotional challenge in healthy female participants. Investigation of these issues in non-clinical individuals has implications for understanding changes associated with clinical anxiety.

Until relatively recently, the main focus of studies investigating the neural correlates of anxiety has been on emotional reactivity and the amygdala (AMY), although the role of other emotion processing brain regions, such as the ventro-lateral prefrontal cortex (vlPFC), the medial prefrontal cortex (mPFC), and the insula has also been investigated [1]. Investigations of the role of anxiety-related individual differences showed that increased reactivity to potential threat conveyed by socially relevant stimuli (e.g., angry faces) is associated with exacerbated activity in AMY in both clinical patients with social anxiety disorder (SAD) (linked to individual variations in the severity of the symptoms) [2], [3], [4], [5], and in non-clinical individuals (linked to variations in the level of anxiety) [6], [7], [8], [9]. This suggests that altered functioning of AMY is not only disorder specific, but could also be observed in individuals who may be prone to develop anxiety disorders [9], [10].

Unlike the brain regions associated with emotion processing, the role of cognitive control brain regions, such as the prefrontal cortex (PFC), in studies of anxiety has been largely neglected [11], although disrupted cognitive control in anxiety has been acknowledged [12], [13]. This might be in part due to the fact that most of the studies mentioned above used relatively simple tasks involving facial expressions that convey criticism and/or negative feedback (e.g., angry, contemptuous faces) to study the neural correlates of emotion processing in anxiety, and only a small number of studies have examined the effects of anxiety on cognitive/executive control brain areas during performance in cognitively demanding tasks. The few recent neuroimaging studies that investigated the neural correlates mediating the alterations in cognitive processing in anxiety reported an under recruitment of dorsal cognitive/executive brain regions including the lateral PFC and dorsal anterior cingulate cortex (ACC) [3], [11], [14]. For instance, Bishop et al. (2004) reported reduced recruitment of the lateral PFC during a lower-level perceptual task involving processing of task-irrelevant emotional face distracters, thus suggesting that increased reactivity to socially relevant emotional cues may impair cognition by distracting anxious individuals from focusing on ongoing goal-relevant tasks [11]. However, given the absence of clear behavioural differences in the impact of emotional vs. neutral distracters, it is not clear whether activity in these brain regions reflects actual impairment in cognitive performance or changes in subjective experience linked to individual variation in personality traits affecting sensitivity to emotional distraction.

Although evidence from clinical and non-clinical investigations suggests that emotional hyper-reactivity and altered cognitive control are mediated by exaggerated response in emotion brain regions and under recruitment of cognitive control regions, little is known about how anxiety influences emotion-cognition interactions [15]. Specifically, the brain mechanisms mediating the disruptive effects of anxiety-related stimuli on higher order cognitive functions and their sensitivity to individual variations in both subjective and objective indices of behaviour remain unclear. Therefore, the main goal of the present investigation was to examine the brain mechanisms mediating the subjective and objective impact of transient anxiety-inducing distracters on cognitive performance, and the role of individual variations in trait anxiety affecting the sensitivity to emotional distraction. In the context of the current investigation, the *subjective* aspect of the impact of emotional distraction refers to its overall perceived effect (compared to that of the non-emotional distraction), regardless of whether it actually impaired cognitive performance or not. On the other hand, the *objective* aspect refers to the actual impact of emotional distraction, as quantified by assessments of working memory performance in the presence of emotional vs. non-emotional distraction. This conceptualization is consistent with evidence pointing to dissociable neural correlates for controlling the *feeling* of being distracted vs. controlling the *actual* impact of emotional distraction [16].

Of particular relevance for the present investigation are studies that examined the impact of transient emotional distraction on performance in tasks involving higher-level cognitive processes. For instance, in a series of studies conducted by Dolcos and colleagues, the neural correlates mediating emotion–cognition interactions were investigated using a paradigm where emotional task-irrelevant distracters were presented during the delay interval of a working memory (WM) task [16], [17], [18]. The main finding of these studies was that the impairing effect of emotional distraction was linked to opposing patterns of activity in ventral affective and dorsal executive brain regions. Specifically, emotional distracters enhanced activity in emotion processing regions, such as AMY, vlPFC, and medial PFC, while disrupting delay activity in dorsal executive brain regions, such as the dorso-lateral PFC (dlPFC) and the lateral parietal cortex (LPC). Given the role of the latter brain regions in attentional processes and active maintenance of goal-relevant information in WM [19], [20], [21], [22], these findings suggest that activity in the affective and executive neural systems is strongly interconnected, in that increased activity in the ventral affective regions disrupts activity in the dorsal system and results in cognitive impairment. It is not clear, however, whether similar effects are observed with specific emotions (e.g., anxiety), and whether these differences are linked to individual differences in personality traits indexing specific aspects of emotion processing (e.g., trait anxiety).

It should also be noted that changes in the brain regions reviewed above may reflect not only the impact of emotional challenge, but also the engagement of cognitive control operations needed to cope with the presence of emotional distraction [16]. However, to our knowledge there is no evidence that links such dissociable patterns of responses reflecting the detrimental impact of emotional distraction vs. the engagement of coping mechanisms to individual differences. Therefore, the second goal of the present study was to distinguish between patterns of brain activity reflecting these opposing effects, in brain regions associated with emotion, perceptual, and cognitive control processing, and to investigate their link to individual differences in trait anxiety and cognitive performance.

The present study addressed these issues by using an adapted version of our WM task with distraction [18] that allowed investigation of the neural mechanisms that mediate cognitive interference by specific transient anxiety-inducing distracters, in conjunction with behavioural measures that assessed the effect of individual variations in both trait anxiety and cognitive performance. Brain activity was recorded using event-related fMRI while healthy participants performed this WM task, and behavioural assessment involved measures indexing the subjective and objective impact of distraction on cognitive performance, as well as measures of personality traits (i.e., trait anxiety). To distinguish between brain responses reflecting the impact of vs. coping with emotional distraction, brain-behavioural correlations were calculated between activity in response to the transient anxiety-inducing emotional distraction and the behavioural measures.

We made the following three predictions. First, we predicted that processing of anxiety-inducing emotional distracters would be associated with opposing patterns of brain activity in affective and executive brain regions. Second, we predicted that activity in these regions would be sensitive to individual variation in both trait anxiety and WM performance. Third, we also predicted that responses reflecting the detrimental impact of emotional distraction vs. the engagement of defensive mechanisms to cope with distracting emotions would be associated with opposing changes of activity in similar but dissociable brain regions.

## Materials and Methods

### Ethics statement

The experimental protocol was approved by the Health Research Ethics Board at University of Alberta and all subjects provided written informed consent.

### Subjects

Eighteen healthy young (18–33 years of age; average = 22.5; SD = 4.25) right-handed adults participated in the study. We restricted our study to female participants for the following three main reasons: 1. To maintain homogeneity of the subject sample, as available evidence shows that that women and men differ in terms of both general emotional reactivity [23], [24], [25] and emotion regulation [26], [27], [28]; 2. To specifically target the subject population that is more prone to develop affective disorders, as suggested by evidence of greater lifetime prevalence of mood and anxiety disorders in women (i.e., nearly two times higher than in men) [29], [30]; 3. To allow more direct comparison with findings from similar previous investigations [17], [18]. Data from two subjects were excluded from analyses because of incompleteness (e.g., due to missing runs); hence, analyses reported are based on behavioural and brain imaging data from sixteen participants (average age = 22.7; SD = 4.47).

### Stimuli

**Figure 1:**
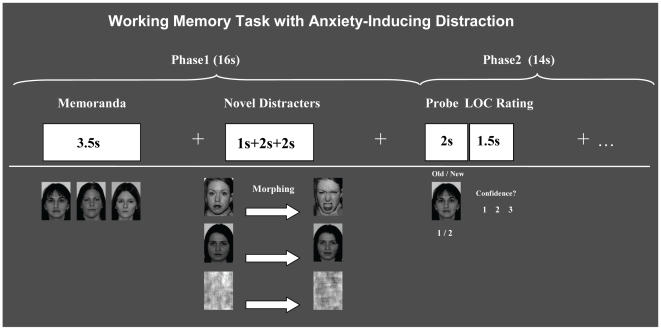
Diagram of the WM Task with Anxiety-Inducing Distraction. Functional magnetic resonance imaging (fMRI) data were recorded while subjects performed a working memory (WM) task for faces, with distraction presented during the delay interval between the memoranda and probes. To increase the impact, the novel distracters were morphed. The WM performance was measured using a recognition memory task, in which participants indicated by pushing a button whether single-face probes were part of the memoranda (*Old* = 1) or not (*New* = 2), and then they indicated the level of confidence (LOC) in their responses by pushing one of three buttons (1 = Low, 2 = Medium, 3 = High). All stimuli were presented in colour. Written informed consent for photograph publication was obtained for all faces illustrated in the figure that are not part of the standardized NimStim Face Stimulus Set.

Subjects performed a modified version of our delayed-response WM task with distraction [18], adapted to fit the purpose of the present investigation (see Figure 1). The memoranda consisted of sets of three human faces (50% females/50% males), chosen to maximize the similarities and hence make the task more difficult. The distracters were presented during the delay interval between the memoranda and probes, and consisted of morphed anxiety-inducing angry faces, neutral faces, and scrambled faces (50% of the distracters were females and 50% were males). Dynamic (morphed), as opposed to static, facial stimuli were used in order to induce responses closer to real-life social interactions; morphing was performed using Winmorph (http://www.debugmode.com/winmorph/). The scrambled faces had the same average spatial frequency and luminance as the meaningful faces, and thus served as no-distraction perceptual controls. Facial stimuli were selected from the set used in our previous studies [17], [18], and were complemented with stimuli from other sources (i.e., the NimStim Face Stimulus Set, http://www.macbrain.org/resources.htm) [31]. A total of 90 experimental trials, identified based on the type of distracters (30 with angry faces, 30 with neutral faces, and 30 with scrambled faces) were involved. All stimuli were presented in color.

### Experimental Procedures

The pool of 90 trials was divided in 6 sets of 15 trials (5 angry, 5 neutral, and 5 scrambled faces per set) which were randomly assigned to 6 experimental blocks/runs. To avoid induction of longer-lasting effects, the trials within each block were pseudo-randomized, so that no more than two consecutive trials of the same type were presented. To prevent possible biases resulted from using the same run order, participants were assigned different run orders, with a total of 6 different run orders being involved. As illustrated in Figure 1, each trial started with the presentation of face memoranda (3.5 s), which subjects were instructed to encode and then maintain into WM during the delay interval between the offset of the memoranda and the onset of the memory probe (12.5 s). Presentation of novel distracters started 2.5 s after the offset of the memoranda, and occurred for a total time of 5 s. All distracters started as static stimuli (either neutral or scrambled), then after a short delay (1 s) they morphed for a 2 s period, which was followed by another static presentation of the final morphed faces (2 s). Half of the initially neutral faces morphed into angry faces with mouth open, while the other half morphed into neutral faces with mouth open (see Figure 1). Also, half of the initially scrambled faces morphed into the corresponding scrambled angry faces, whereas the other half morphed into the corresponding scrambled neutral faces.

Subjects were instructed to look at the distracters but maintain focus on the WM task. At the end of the delay interval, a single probe face was presented for 2 s, and the subjects' task was to indicate by pressing a button whether the probe was previously included as one of the three faces in the current memorandum or whether it was a new face (50% probes were *Old* and 50% were *New*). Subjects were instructed to make quick and accurate responses while the probes were on the screen. Then, they had 1.5 s to rate the level of confidence (LOC) of their responses using a 3-point Likert scale (1 = lowest, 3 = highest). The LOC rating was followed by a 10.5 s inter-trial interval to allow the hemodynamic response to return to baseline; during this time, the subjects were instructed to relax and refrain from doing anything systemically that could potentially affect the inter-trial baseline signal (e.g., counting). The total length of each trial was 30 s. Following scanning, subjects performed two consecutive long-term recognition memory tasks that tested their episodic memory for the memoranda and the distracters (not reported). The recognition memory tasks were followed by an emotional rating task, in which subjects had to rate how angry they perceived the emotional and neutral distracters, using a 9-point Likert scale (1 =  not angry at all; 9 = very angry). These ratings were assessed to confirm that the angry faces were perceived as more emotional than neutral faces.

### Personality and Affective State Measures

The present participant sample is part of a larger group in which various aspects of behaviour (i.e., related to cognition, emotion, and personality) are assessed in order to investigate the role of individual differences in cognitive-affective interactions. For the purpose of the present investigation, we mainly focused on personality assessments of both general and specific anxiety traits. General trait anxiety was measured using the trait scale of the State Trait Anxiety Inventory (STAI-T) [32], and the specific trait anxiety linked to social behaviour was measured using the Liebowitz Social Anxiety Scale (LSAS) [33], [34]. Given the specific nature of the transient emotional distracters used in the present study (i.e., angry faces expected to induce social anxiety) and previous evidence showing that the neural response to angry and fearful faces may be specifically influenced by trait social anxiety [4], we decided to also specifically assess social trait anxiety, in addition to assessing general trait anxiety. By using these two measures of anxiety, one indexing the level of general (non-specific) anxiety and the other indexing the level of specific (social) anxiety, the present study sought to investigate potential dissociable effects linked to emotion-cognition interactions in the presence of anxiety-inducing distraction. To assess the present general and anxiety-related emotional state, participants also completed the state scale of the Positive and Negative Affective Schedule (PANAS-S) [35] and the state scale of STAI (STAI-S) [32], both at the beginning and at the end of the study.

### Imaging Protocol

Scanning was conducted on a 1.5T Siemens Sonata scanner. After the sagittal localizer and the 3D magnetization prepared rapid acquisition gradient echo (MPRAGE) anatomical series (TR = 1600 ms; TE = 3.82 ms; number of slices = 112; voxel size = 1×1×1 mm^3^), series of 28 functional slices (voxel size = 4×4×4 mm^3^) were acquired axially using an echoplanar sequence (TR = 2000 ms; TE = 40 ms; FOV = 256×256 mm), thus allowing for full-brain coverage.

### Behavioural Data Analyses

Responses in the WM task were classified in one of the four categories derived from signal detection theory [36]: (1) *Hits*, corresponding to memorandum faces correctly classified as *Old* (i.e., as being part of the memoranda), (2) *Misses*, corresponding to memorandum faces incorrectly classified as *New*, (3) *Correct Rejections* (CRs), corresponding to new faces correctly classified as *New*, and (4) *False Alarms* (FAs), corresponding to new faces incorrectly classified as *Old*. For more accurate assessment of WM performance, corrected recognition scores (% Hits - % FAs) were also calculated for each participant. Differences in WM performance among the three trial types (emotional vs. neutral vs. scrambled) were assessed using repeated measures ANOVAs. Relationships between WM performance and trait anxiety were assessed using correlation analyses between WM performance and the STAI-T and LSAS scores. Finally, differences in general and anxiety-related affective state were also assessed using *t* statistics, which compared the PANAS-S and STAI-S scores before and after the study.

### fMRI Data Analyses

Statistical analyses were preceded by the following pre-processing steps (performed with SPM2 - Statistical Parametric Mapping): TR alignment, motion correction, co-registration, normalization, and smoothing (8 mm^3^ Kernel). Data analysis used in-house custom MATLAB scripts and involved both whole-brain voxel-wise and region of interest (ROI) analyses [18], to compare brain activity associated with the conditions of interest (e.g., trials with anxiety-inducing angry distracters vs. trials with neutral distracters). For subject-level analyses, the fMRI signal was selectively averaged in each subject's data as a function of trial type (i.e., angry, neutral, and scrambled distracters) and time point (one pre-stimulus and 13 post-stimulus onset time points), using custom MATLAB software, and pair-wise *t* statistics for the contrast of interest (e.g., anxiety-inducing vs. neutral distracters) were calculated for each subject. No assumption was made about the shape of the hemodynamic response function. This method has proven particularly effective in dissociating responses (reflected in both activation and *de*activation) produced by our WM task with emotional distraction, in both healthy and clinical groups [17], [18], [37]. Moreover, this method also allows finer comparisons of the MR signal on a time TR-by-TR basis. Individual analysis produced whole-brain average and activation *t* maps for each condition, contrast of interest, and time point. The outputs of subject-level analyses were used as inputs for second-level random-effects group analyses. All analyses focused on effects observed at the time point within the 14–18 s period after the memoranda onset, when the differential effects of the distracters on activity during the delay period were most evident [18].

The first main goal of the present investigation was to examine brain activity in the ventral and dorsal neural systems in response to transient anxiety-inducing distraction, and how it is modulated by individual variations in both trait anxiety and actual cognitive performance. The second main goal was to look for evidence dissociating responses that reflect the engagement of defensive mechanisms to cope with distracting emotions from those reflecting the detrimental impact of emotional distraction, that are linked to individual differences in trait anxiety and cognitive performance. These goals were accomplished by first identifying the brain regions whose activity was specifically sensitive to the presence of anxiety-inducing distracters. Then, activity in these regions was tested for co-variation with trait anxiety and WM scores, to identify responses reflecting the detrimental impact of emotional distraction vs. the engagement of defensive mechanisms to cope with distracting emotions. These analyses are described in detail below.

To identify the brain regions whose activity was sensitive to the presence of anxiety-inducing distracters, we investigated brain activity in ventral and dorsal neural systems previously identified as sensitive to the presence of general negatively-valenced distraction [18]. For this, we employed conjunction analyses consisting of the following two steps. First, *t* maps contrasting the emotional distracters to both the neutral and the scrambled distracters were independently calculated. Then, conjunction maps were obtained by masking with each other the statistical maps resulted from the first step. For activity in the ventral emotional system, separate *t* maps were computed to identify voxels where emotional distracters produced greater activity than both the scrambled and the neutral distracters (emotional > scrambled & emotional > neutral). Then, these statistical maps were inclusively masked with each other, to identify regions of overlap [(emotional > scrambled) ∩ (emotional > neutral)] showing *in*creased activations specific to the emotional distracters. Similarly, to identify activity in the dorsal executive regions that was sensitive to the presence of emotional distracters, separate *t* maps were first computed to identify voxels where emotional distracters produced reduced activity compared to both scrambled and neutral distracters (emotional < scrambled & emotional < neutral). Then, these statistical maps were inclusively masked with each other, to identify overlapping regions [(emotional < scrambled) ∩ (emotional < neutral)] showing *de*creased activations that were specific to emotional distracters. A threshold of p<0.01 was used for the contrasts between the most dissimilar conditions (emotional > scrambled and emotional < scrambled) and a threshold of p<0.05 was used for contrasts between more similar conditions (emotional > neutral and emotional < neutral). Hence, the joint threshold of the resulting conjunction maps was p<0.0005 [38]. An extent threshold of 10 contiguous voxels was used in each of the contributing maps.

To further investigate whether activity in the brain regions sensitive to transient anxiety-inducing distraction is modulated by individual variations in trait anxiety and actual cognitive performance, we performed triple conjunction analyses. Two of the statistical maps involved in the triple conjunctions were obtained using the same procedure as described above. The third map consisted of a correlation map identified by co-varying average brain activity in response to task-irrelevant distraction with individual scores indexing trait anxiety and WM performance. Thus, to identify brain regions within the ventral and dorsal system whose activity is specific to emotional distraction and sensitive to individual variations in trait anxiety, we performed a triple conjunction analysis between (1) activation maps identifying differential (higher or lower) activity for the emotional compared to scrambled distracters (emotional > scrambled, in the ventral system, and emotional < scrambled, in the dorsal system), (2) activation maps identifying differential activity for the emotional compared to neutral distracters (emotional > neutral, in the ventral system, and emotional < neutral, in the dorsal system), and (3) correlation maps identifying co-variations between brain activity in the presence of distracters and scores indexing personality traits related to both general and social anxiety (as measured with STAI-T and LSAS, respectively).

A similar procedure was used to identify brain regions whose activity is specific to emotional distraction and sensitive to individual variations in WM performance, with the only difference being that the third statistical map contributing to the triple conjunction analyses consisted of a correlation map identifying co-variations between brain activity in the presence of distracters and scores indexing WM performance (i.e., corrected recognition scores  =  % Hits minus % False Alarms). For all triple conjunctions, an intensity threshold of p<0.05 was used in each of the contributing maps, and hence the joint threshold of the resulting conjunction maps was p<0.0005 [38]. An extent threshold of 10 contiguous voxels was used in each of the contributing maps.

Importantly, these analyses also allowed identification of patterns of co-variation linked to individual differences in trait anxiety and cognitive performance that dissociate responses reflecting the engagement of defensive mechanisms to cope with distracting emotions from those reflecting the detrimental impact of emotional distraction. For instance, increased activity to emotional distraction in perceptual brain regions, coupled with negative co-variation with WM performance, would be indicative of a detrimental impact of emotional distraction that reflects bottom-up effects. On the other hand, positive co-variation of activity in cognitive control brain regions, in response to emotional distraction, with WM performance would be indicative that activity in those regions reflects the engagement of top-down mechanisms to cope with distraction.

Activity in the main ventral affective and dorsal executive brain regions identified by the whole-brain voxel-wise analyses was subject to further confirmatory investigations, using a functional region of interest (ROI) approach. This involved extraction of the MR signal, for each subject, condition, and time point, from voxels identified by the group conjunction analyses. Then, across-subjects correlations between the extracted MR signal (expressed in percent signal change) recorded at the delay peak time point (i.e., 14–16 s following the memoranda onset) and the individual scores for trait anxiety (STAI-T/LSAS scores) and WM performance (corrected recognition scores) were calculated. The main goals of these additional investigations were to check whether some of the effects identified by the voxel-based correlation analyses were driven by outliers, and to test the specificity of the co-variations (i.e., by calculating the significance of the differences between correlation coefficients for emotional and neutral distracters) [39]. The signal extracted from the ROIs was also used for illustration purposes (i.e., in the creation of figures).

Finally, as a general rule analyses not involving measures of WM performance were performed on fMRI data from all trials (i.e., 30 per condition). This was based on the fact that participants systematically evaluated the angry face distracters as being more emotional and distracting (as assessed with ratings and during debriefing), regardless of their actual impact on WM performance. However, the perceived subjective effect of emotional distraction may differ from its actual impact on cognitive performance [16]. Therefore, analyses aimed at identifying brain activity sensitive to individual variation in the objective measure of cognitive performance focused on trials reflecting the actual behavioural impact of distraction on WM performance, and thus involved analyses of fMRI data from correct trials only (i.e., Hits and CRs).

## Results

### Behavioural Results

#### Working Memory Performance

**Figure 2:**
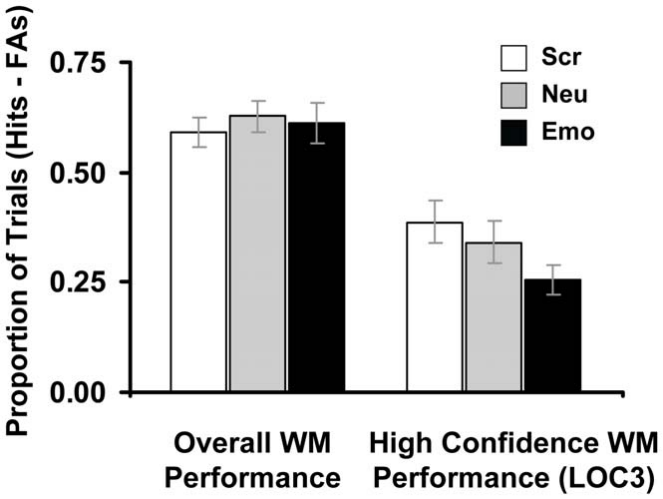
The detrimental effect of transient anxiety-inducing distraction on WM performances was reflected in responses with the highest level of confidence (LOC3). A two-way ANOVA on corrected recognition scores (% Hits – % FAs) yielded a significant level of confidence (LOC1, LOC2, LOC3) x distracter type (emotional, neutral, scrambled) interaction (F [4], [60]  = 8.57; p<0.00002), and post-hoc analyses showed that the emotional distraction had an impairing effect only on LOC3, with the emotional distracters being associated with lower level of performance compared to both neutral (p<0.02) and scrambled distracters (p<0.0005). Emo = Emotional Distracter; Neu  =  Neutral Distracters; Scr  =  Scrambled Distracters; FAs  =  False Alarms; WM  =  Working Memory. Error bars represent standard errors.

Analyses of WM results showed that the detrimental effect of anxiety-inducing distracters was reflected only in responses with the highest level of confidence. Overall, participants correctly identified probes that were part of the memoranda (Hits) on 68.74% (SD = 17.41) of the trials with anxiety-inducing distracters, 74.18% (SD = 11.96) of the trials with neutral distracters, and 73.75% (SD = 12.04) of the trials with scrambled distracters. A one-way ANOVA yielded a non-significant main effect of distracter type, thus suggesting that the overall WM performance was equivalent in all three trial types; the same results were obtained with the corrected recognition scores (%Hits - %FAs) (see Figure 2). However, further investigation of the emotion impact on WM performance according to the level of confidence (LOC; 1 = lowest, 3 = highest) identified a significant detrimental effect of angry face distracters on the responses associated with the highest level of confidence (i.e., LOC3) (Figure 2). A two-way ANOVA on items correctly identified as old yielded a significant level of confidence (LOC1, LOC2, LOC3) x distracter type (emotional, neutral, scrambled) interaction (F [4], [60]  = 6.24; p<0.0003). Second, post-hoc analyses showed that the emotional distraction had an impairing effect only on LOC3, with the emotional distracters being associated with lower level of performance compared to both neutral (p<0.005) and scrambled distracters (p<0.0002). These findings were also confirmed by similar ANOVA and post-hoc analyses on the corrected recognition scores (Figure 2). Because the detrimental effect of anxiety-inducing distraction affected only the LOC3 responses, analyses involving WM performance focused only on these responses.

#### Emotional Ratings and Personality Measures

As expected, subjects rated the angry faces as being more emotional than the neutral faces; the average scores for emotional intensity (1 = lowest, 9 = highest) as rated by the participants were 7.52 (SD = 0.67) for the angry face distracters and 2.07 (SD = 0.72) for the neutral face distracters. This was confirmed by a pair-wise comparison of the emotional rating scores for the angry and neutral faces (T _(15)_  = 24.42; p<0.0001). Also confirming that our manipulation worked in inducing anxiety, participants had significantly higher levels of state anxiety after the completion of the task compared to the beginning of the study, as identified by significant pre- vs. post-task differences in the STAI-S scores (T _(15)_  = −2.98; p<0.01). In addition, participants also had significantly lower levels of state positive affect after the completion of the task, as identified by significant pre- vs. post-task differences in the positive state scores of the PANAS-S scale (T _(15)_  = 3.13; p<0.01). The scores for the state-related measures (STAI-S and PANAS-S) and the trait measures related to anxiety (STAI-T and LSAS) are presented in Table 1.

**Table 1:** Trait and/or State Scores for General Affect (PANAS), General Anxiety (STAI) and Specific Social Anxiety (LSAS).

LSAS	45.81 (20.06)
STAI-Trait	40.75 (8.10)
PANAS Trait (positive)	31.43 (7.31)
PANAS Trait (negative)	14.25 (3.84)
STAI-State pre	33.19 (7.53)
STAI-State post	36.5 (6.09)**
PANAS State pre (positive)	28.69 (6.61)
PANAS State post (positive)	25.56 (5.69)**
PANAS State pre (negative)	12.00 (1.97)
PANAS State post (negative)	11.81 (1.17)

All measures are reported as mean (SD).

**Significant pre-post study differences (p<0.01).

#### Relationship between Individual Variations in Anxiety and WM Performance

The present study also identified a positive correlation between general trait anxiety and WM performance for the condition where the emotional distraction had a detrimental effect (i.e., the LOC3 responses). Specifically, subjects self-reporting higher levels of general trait anxiety were also better at correctly identifying old items as being *Old* for the emotional distracters (r = 0.62; p<0.01), but not for the neutral (r = 0.38; p>0.1) or scrambled (r = 0.35; p>0.1) distracters. These findings suggest that participants with higher level of general trait anxiety benefited in performing the WM task with anxiety-inducing distraction. These numerical differences were also confirmed by correlations based on the corrected recognition scores, although these correlations were also significant for the neutral and scrambled distracters (r = 0.64, p = 0.004 for emotional; r = 0.44, p = 0.04 for neutral; and r = 0.45, p = 0.04 for scrambled distracters). No significant correlations between the trait social anxiety as measured with LSAS and LOC3 performance were found.

### fMRI Results

#### 1. Differential Patterns of Activity in Ventral and Dorsal Neural Systems to Anxiety- Inducing Distraction

As expected, anxiety-inducing distracters produced dissociable patterns of activity in the ventral affective and dorsal executive neural systems (see Table 2). Specifically, when compared with both neutral and scrambled face distracters, angry faces evoked stronger activity in typical brain regions involved in affective processing, including AMY and the ventro-medial PFC (vmPFC), or sensitive to emotional stimulation, such as the fusiform gyrus (FG). By contrast, angry face distracters evoked strong *de*activations in typical brain regions involved in cognitive control and attentional processes, including the dlPFC, the dorso-medial PFC (dmPFC), and the LPC. These findings replicate and extend the effects produced by emotional distraction inducing general negative affect [18], by showing that similar effects are produced by specific anxiety-inducing distraction.

**Table 2:** Differential Effect of Emotional Distraction in Ventral vs. Dorsal Neural Systems.

Brain Regions		BA	Talairach Coordinates (*xyz*)			T values	Time (TR)
Showing *In*creased Activity (Emo > Scr & Emo > Neu)							
vmPFC	R Medial Frontal Gyrus	BA 10	4	50	−6	5.67	9
TOC	L Fusiform Gyrus	BA 37	−44	−59	−7	5.22	9
	L Inferior Occipital Gyrus	BA 19	−40	−72	−3	7.72	9
	R Fusiform Gyrus	BA 37	40	−51	−8	8.49	9
	R Inferior Occipital Gyrus	BA 19	40	−70	−3	8.62	9
	R Middle Occipital Gyrus	BA 19	48	−77	11	6.83	9
Amygdala	R Amygdala		20	−8	−13	4.13	9
Showing *De*creased Activity (Scr > Emo & Neu > Emo)							
dlPFC	R Superior Frontal Gyrus	BA 10	24	51	12	4.96	9
	R Middle Frontal Gyrus	BA 9	40	32	28	5.22	9
	L Middle Frontal Gyrus	BA 46	−40	36	24	6.80	9
	L Middle Frontal Gyrus	BA 8	−40	25	43	6.62	9
dmPFC	L Medial Frontal Gyrus	BA 6	−4	−9	59	7.52	9
	L Superior Frontal Gyrus	BA 6	−4	6	48	6.50	9
LFC	R Precentral Gyrus	BA 6	55	−3	11	4.29	9
	L Precentral Gyrus	BA 6	−59	−3	11	5.33	8
Insula	L Insula	BA 13	−40	−19	5	3.46	9
LPC	R Inferior Parietal Lobule	BA 40	40	−48	50	4.36	9
	R Postcentral Gyrus	BA 43	51	−19	16	4.58	9
	L Postcentral Gyrus	BA 43	−51	−15	15	7.99	8
MPC	L Precuneus	BA 7	−8	−51	58	6.15	9
LTC	R Superior Temporal Gyrus	BA 42	59	−26	16	4.84	9
	L Superior Temporal Gyrus	BA 22	−51	−15	8	11.39	8
	L Middle Temporal Gyrus	BA 20	−63	−43	−11	6.88	9
MOC	R Cuneus	BA 7	12	−68	33	5.89	9
	L Cuneus	BA 18	0	−73	15	3.89	9

vmPFC  =  Ventro-medial Prefrontal Cortex; TOC  =  Temporo-Occipital Cortex; dlPFC  =  Dorso-lateral Prefrontal Cortex; dmPFC  =  Dorso-medial Prefrontal Cortex; LFC  =  Lateral Frontal Cortex; LPC  =  Lateral Parietal Cortex; MPC  =  Medial Parietal Cortex; LTC  =  Lateral Temporal Cortex; MOC  =  Medial Occipital Cortex; BA  =  Brodmann Area; *x, y, z* denote coordinates in the Talairach space [74]; TR  =  Repetition Time; Emo  =  Emotional Distracter; Neu  =  Neutral Distracters; Scr  =  Scrambled Distracters.

#### 2. Co-variation of Brain Activity with Individual Differences in Anxiety and WM Performance

**Figure 3:**
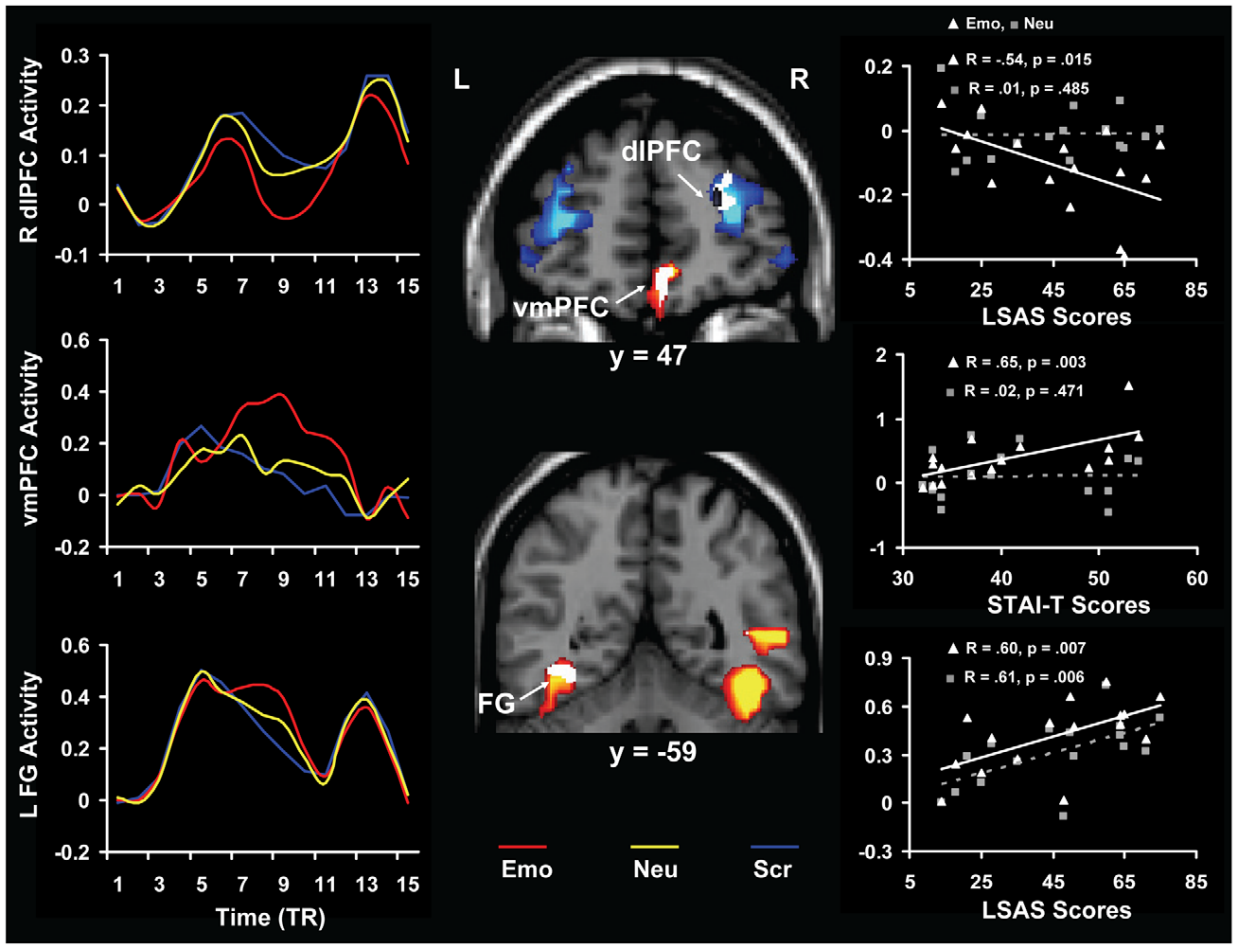
Opposite patterns of activity and co-variation in the ventral vs. dorsal neural systems in the presence of anxiety-inducing distracters. Consistent with a bottom-up effect of emotional distraction on brain activity, ventral regions associated with perception (FG, bottom panels) and experiencing of emotion (vmPFC, middle panels) showed *in*creased overall activity (red blobs) and positive correlations with anxiety scores (white blobs within the red blobs), whereas dorsal regions associated with executive functions (e.g., dlPFC, top panels) showed *de*creased overall activity (blue blobs) and negative correlations with anxiety scores (white blobs within the blue blobs). Because in the FG the correlation was specific for social anxiety, whereas in the vmPFC it was larger for general anxiety (see Table 3), the scatterplots on the right side of the figure are based on the corresponding correlations of the signal extracted from the ROIs with the LSAS and STAI-T scores, respectively. In the vmPFC, the positive correlation was specific for the emotional distraction - i.e., the correlation was significant for the emotional but not for the neutral distracters, and the difference between these two correlations was also significant (see Table 3). In the dlPFC, although in the whole ROI (white blob) the negative correlation was not statistically greater for the emotional distracters (see Table 3), a restricted area within this ROI (the black blob within the white blob) showed specificity for the emotional distracters. As illustrated in the top right scatterplot, the correlation was significant for the emotional but not for the neutral distracters, and the difference between these two correlations was also significant (t = −1.92; p = 0.04). A similar pattern was also observed in the dmPFC (not shown). The activation and conjunction/correlation maps are superimposed on high resolution brain images displayed in coronal views (y indicates the Talairach coordinate on the anterior-posterior axis of the brain). The line graphs on the left side panels illustrate the time courses of the fMRI signal, as extracted from the ROIs meeting the triple conjunction criteria (the white blobs), on a TR-by-TR basis (1 TR = 2 seconds). FG  =  Fusiform Gyrus; vmPFC  =  Ventro-medial Prefrontal Cortex; dlPFC  =  Dorso-lateral Prefrontal Cortex; L =  Left; R =  Right; TR  =  Repetition Time.

The present study also identified effects of individual variations in trait anxiety and WM performance on brain activity in response to anxiety-inducing distraction. First, activity in the same ventral and dorsal regions that were sensitive to the presentation of transient anxiety-inducing distracters was also differentially modulated by individual variations in general and social trait anxiety, as measured with STAI-T and LSAS, respectively (see Table 3 and Figure 3). For instance, activity in the left visual cortex (including the left FG, BA 37) and vmPFC was positively correlated, and activity in the right dlPFC and dmPFC was negatively correlated with anxiety scores. Notably, with the exception of activity in the left FG, all of these brain-behaviour co-variations were specific or numerically greater for the anxiety-inducing angry face distracters (see Table 3). Also, while overall similar effects were observed for general (Table 3A) and social anxiety (Table 3B) in these brain-behaviour co-variations, exceptions were also noted. For instance, in the left FG, the effect was specific to social anxiety, whereas in the vmPFC the effect was larger for general anxiety (Table 3).

**Table 3:** Correlations between Brain Activity and A. General Trait Anxiety (STAI-T scores) and B. Specific Social Trait Anxiety (LSAS scores).

Brain Regions		BA	Talairach Coordinates			Correlations (r values)			Emo r *vs.* Neu r
			*x*	*y*	*z*	Emo	Neu	Scr	
A.									
Showing *In*creased Activity and *Positive* Correlation									
vmPFC	L Medial Frontal Gyrus	BA 10	0	50	−9	0.65***	0.02	0.02	t = 3.16***
Showing *De*creased Activity and *Negative* Correlation									
dlPFC	R Middle Frontal Gyrus	BA 10	24	51	16	−0.67***	−0.13	−0.02	t = −2.25*
LFC	L Precentral Gyrus	BA 6	−59	−2	33	−0.70***	−0.52*	−0.03	t = −1.04
dmPFC^1^	L Medial Frontal Gyrus	BA 6	−4	2	48	−0.58**	−0.30	−0.13	t = −0.97
B.									
Showing *In*creased Activity and *Positive* Correlation									
vmPFC^1^	L Medial Frontal Gyrus	BA 10	−4	42	−9	0.43*	−0.24	−0.27	t = 2.56*
TOC^1^	L Fusiform Gyrus	BA 37	−38	−58	−6	0.60**	0.61**	0.34	t = −0.13
Showing *De*creased Activity and *Negative* Correlation									
dlPFC	R Middle Frontal Gyrus	BA 10	32	40	24	−0.62**	−0.41†	−0.18	t = −1.17
LFC	L Precentral Gyrus	BA 6	−59	−2	33	−0.65***	−0.10	−0.06	t = −2.90**
dmPFC	L Medial Frontal Gyrus	BA 6	−8	6	48	−0.63***	−0.47*	−0.25	t = −0.72

The r values correspond to the co-variation between the signal extracted from the whole ROIs (as identified by the triple conjunction), and anxiety scores. The statistical differences between the effects for emotional and neutral distracters, as tested using the r-to-t transformation for comparison of overlapping correlations [39], are noted in the last column.

*Significance at p<0.05.

**Significance at p<0.01.

***Significance at p<0.005.

† p<0.06.

Note:

1 Effects present at lower extent threshold or absent for general or social anxiety. In the Fusiform Gyrus (FG), the correlation was strong for social anxiety, but absent for general anxiety. In the ventro-medial PFC (vmPFC), the effect was overall larger for general anxiety, but still present for social anxiety at a lower extent threshold (3 voxels). In the dorso-medial PFC (dmPFC) both effects were present, but at a lower extent threshold for general anxiety (9 voxels).

These findings suggest that enhanced trait anxiety is associated with increased sensitivity to transient anxiety-inducing stimulation, which results in enhanced activity in brain regions associated with the perception and experiencing of emotions (FG and vmPFC, respectively), and impaired activity in brain regions associated with the ability to maintain focus on goal-relevant information (dlPFC).

**Figure 4:**
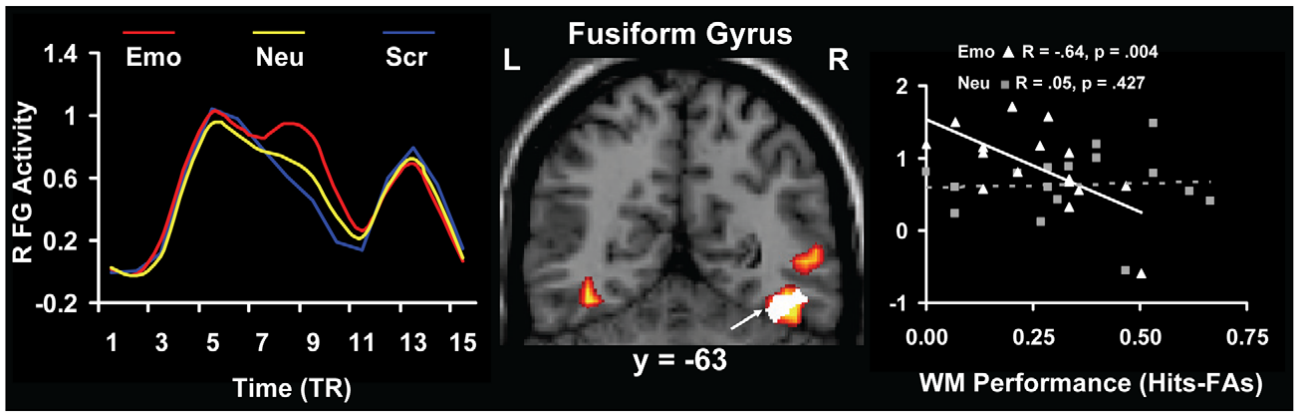
Co-variation between activity in the right fusiform gyrus (FG) and individual differences in WM performance. Consistent with a bottom-up impact of emotional distraction on cognitive performance, the right FG showed increased overall activity (red blob) and negative correlation with the LOC3 WM performance (the white blob within the red blob). The negative correlation, illustrated in the right side scatterplot was specific for the emotional distracters (see Table 4). The middle panel illustrates the activation and correlation maps superimposed on high resolution brain images, displayed in coronal view. The line graph in the left side panel illustrates the time course of fMRI signal, as extracted from the whole ROI meeting the triple conjunction criteria, on a TR-by-TR basis.

Second, to investigate how activity in the ventral and dorsal brain regions is linked to individual differences in the actual cognitive performance, brain-behaviour correlations were performed between activity in brain regions that were more sensitive to the presence of emotional distraction (i.e., brain regions showing *in*creased or *de*creased activity to angry face distracters compared to both the neutral and the scrambled faces) and WM scores. Because the detrimental effect of anxiety-inducing distracters was reflected in the responses associated with the highest level of confidence (LOC3), these analyses involved only the correct LOC3 trials. These analyses identified areas of the right visual cortex, including the right FG (BA 37), whose activity was negatively correlated with WM performance (r = −0.64; p = 0.004) in the presence of emotional distraction (Figure 4). Given that this effect was specific for the anxiety-inducing distracters (i.e., it was significant for the emotional distracters, but not for neutral distracters, and the difference between these two correlations was also significant, see Table 4), these findings suggest that the engagement of these areas may reflect the impairing impact of emotional distraction on WM performance, possibly as a result of enhanced visual processing of anxiety-inducing distracters.

**Table 4:** Correlation between Brain Activity and WM Performance.

Brain Regions		BA	Talairach Coordinates			Correlations (r values)			Emo r *vs.* Neu r
			*x*	*y*	*z*	Emo	Neu	Scr	
Showing *In*creased Activity and *Negative* Correlation									
TOC	R Fusiform Gyrus	BA 37	40	−59	−7	−0.64***	0.05	−0.16	z = −2.06*
	R Middle Occipital Gyrus	BA 18	28	−93	5	−0.48*	0.22	−0.08	z = −1.90*
Showing *De*creased Activity and *Positive* Correlation									
LPFC	L Inferior Frontal Gyrus	BA 10/47	−44	47	−2	0.63***	0.29	−0.22	z = 1.13
dmPFC	L Medial Frontal Gyrus	BA 8	−4	22	47	0.57*	−0.13	0.04	z = 1.98*
LPC	L Postcentral Gyrus	BA 43	−48	−11	19	0.62**	0.04	0.01	z = 1.75*
LTC	L Middle Temporal Gyrus	BA 21	−59	−51	−4	0.63***	−0.04	0.11	z = 1.99*

The r values correspond to the co-variation between the signal extracted from the whole ROIs (as identified by the triple conjunction) and WM performance. The statistical differences between the effects for emotional and neutral distracters, as tested using the r-to-z transformation for comparison of nonoverlapping correlations [39], are reported in the last column. LPFC  =  Lateral Prefrontal Cortex.

*Significance at p<0.05.

**Significance at p<0.01.

***Significance at p<0.005.

#### 3. Patterns of Brain Activity Reflecting the Engagement of Defensive Mechanisms to Cope with Distraction

**Figure 5:**
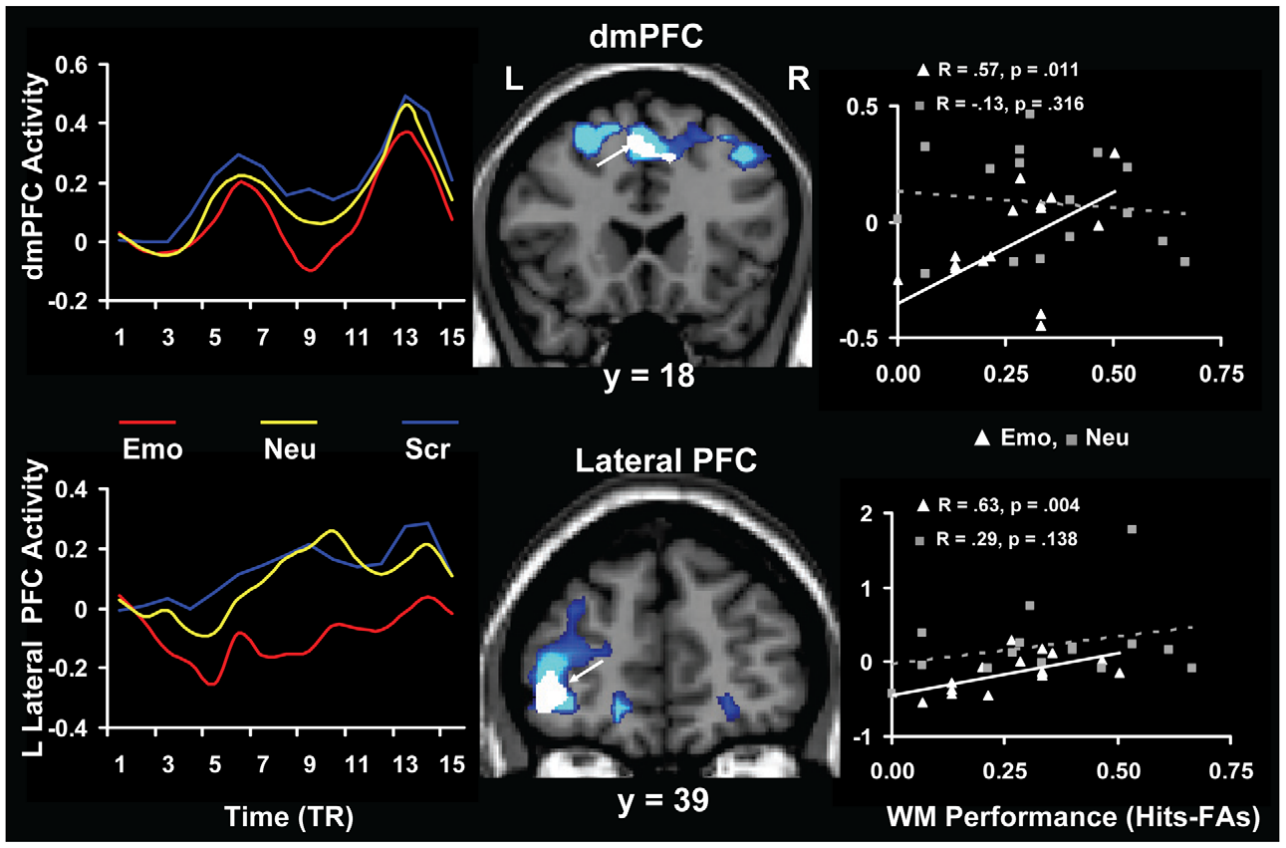
Evidence for the role of the PFC in coping with emotional distraction. Regions in the dorso-medial and left lateral PFC showed positive correlations with the LOC3 WM performance (white blobs within the blue blobs), despite showing overall decreased activity (blue blobs) in the presence of emotional distraction. In both cases, the correlations were significant only for the emotional distracters, and in the dmPFC the correlation for emotional distracter was also statistically greater than for the neutral distracters (see Table 4). The line graphs in the left side panels illustrate the time course of fMRI signal, as extracted from the whole ROIs meeting the triple conjunction criteria, on TR-by-TR. The activation and correlation maps are superimposed on high resolution brain images displayed in coronal views.

The correlation analyses between brain activity and WM scores also identified brain-behaviour relationships that may reflect responses engaged to cope with the presence of anxiety-inducing distraction, as opposed to reflecting a detrimental impact of emotional distraction (see Table 4). For instance, these analyses identified two areas in the dorso-medial (BA 8) and lateral (BAs 10/47) frontal cortex, whose activity was positively correlated with WM performance (r = 0.57; p = 0.011 and r = 0.63; p = 0.004, respectively), in the presence of emotional distraction (Figure 5 and Table 4). In both regions, these effects were significant only for the emotional distracters, and in the dorso-medial PFC the effect was also significantly stronger than for the neutral distracters (see Table 4).

These findings suggest that the effects reflecting the engagement of mechanisms to cope with emotional distraction are present in brain regions associated with cognitive control/executive functions, and that they are expressed in brain-behaviour relationships involving individual differences in cognitive performance.

## Discussion

The present study yielded three main results. First, it extended previous findings of opposing patterns of activity in the ventral affective vs. dorsal executive neural systems produced by distracters inducing general negative emotions (i.e., complex emotionally negative scenes) by showing similar effects with distracters inducing a specific emotion (i.e., transient anxiety-inducing angry faces). Second, the study identified specific brain regions whose activity co-varied with individual differences in trait anxiety and cognitive performance. Third, the present study also identified brain-behaviour relationships consistent with changes in brain activity reflecting responses engaged to cope with the presence of anxiety-inducing distraction, as opposed to reflecting a detrimental impact of emotional distraction. These findings will be discussed in turn below.

### Differential Patterns of Activity in Ventral and Dorsal Neural Systems to Anxiety-Inducing Distraction

Previous investigations using task-irrelevant distracters that induce general negative affect identified opposing patterns of activity in affective and executive regions (*in*creased vs. *de*creased, respectively) [18], which were demonstrated to be specific to emotional distraction [17], [40], [41]. Complementing these investigations, here we show that similar effects are obtained with task-irrelevant distracters inducing specific emotions (i.e., anxiety). These findings are also in line with evidence linking hyperactivity of the ventral system during processing of threat-related cues, both in non-clinically anxious but anxiety-prone participants [9], [10] and in clinically anxious patients [1], [2], [3], [4], [5], [42], [43]. Fewer studies, however, also reported deficient recruitment of activity in the dorsal regions (e.g. dlPFC) in highly anxious non-clinical subjects or in SAD patients [3], [11], particularly in the context of experimental manipulations that simultaneously engage the affective and the executive neural systems. By contrast, the present design in conjunction with personality assessments allowed finer evaluation of activity in the affective and executive systems and of their interactions that were linked to the actual detrimental impact of emotional distraction and the response to cope with it. These aspects will be discussed in detail in the following sections.

### Co-variation of Brain Activity with Individual Differences in Anxiety and WM Performance

One novel aspect of the present study is identification of evidence concerning the role of individual variations in trait anxiety in the interplay between the ventral affective and dorsal executive neural systems in response to transient anxiety-inducing distraction. Although exceptions may be noted [7], previous functional neuroimaging investigations of the relationship between brain activity and anxiety tended to focus separately on emotion [6], [9], [44] or on executive [11], [45] processing regions, or did not use threat related stimuli [14]. Thus, to our knowledge there is no direct evidence concerning the interplay between the emotion and cognition regions as a function of trait anxiety in the context of a cognitively demanding task. The present findings provide this evidence by showing for the first time that individual differences in trait anxiety modulate the interplay between emotion-sensitive and executive control brain regions. Specifically, we show that enhanced levels of trait anxiety are associated with enhanced activity in the FG and greater disruption of activity in dlPFC and dmPFC in response to task-irrelevant anxiety-inducing distracters. Notably, in the FG the positive co-variation was observed only with the trait social anxiety scores, which is consistent with evidence concerning the role of this regions in face processing [46], [47], along with evidence of increased sensitivity of its activity to threatening facial stimuli in patients with SAD [2], [43], [48], and with the way transient social anxiety was induced in the present study (i.e., by angry faces). The dlPFC and dmPFC findings are consistent with recent findings of under recruitment of the prefrontal cortex in anxious individuals [7], [11], [14]. Although activity in these regions was not linked to variations in WM performance, these findings suggest a role of bottom-up sensory-driven mechanisms in the sensitivity to anxiety-inducing distracters, in which increased activity in perceptual areas may impair activity in brain regions responsible for active maintenance of goal relevant information.

In addition to these regions, which are probably part of a basic network sensitive to emotion processing and individual variation in trait anxiety, activity in the vmPFC also deserves further consideration. Compared to the dlPFC response, this region showed opposite patterns of overall activation and co-variation with trait anxiety, which is consistent with previous findings of reciprocal modulations between medial and lateral PFC activity during emotion-cognition interactions [49], [50]. The present vmPFC findings are also consistent with studies linking the medial PFC with various aspects of processing, from general emotion processing [51], to specific self-referential processing [52], [53], [54]. Consistent with the idea that activity in this region might reflect enhanced personal significance of anxiety-inducing angry faces in anxious participants, the response of this region was positively correlated with trait anxiety. This idea is also supported by evidence that activity in a similar medial PFC region, in response to trauma-related negative distracters, was positively correlated with scores indexing the severity of post-traumatic stress disorder symptoms in war veterans, for whom trauma-related distracters are likely to have enhanced personal significance [55]. Thus, further investigation of activity in this region in healthy participants with various degrees of trait anxiety may prove useful in identifying markers indexing individual variations in the susceptibility to affective disorders, in general, and to anxiety-related psychiatric conditions, in particular.

Turning to the objective impact of emotional distraction on WM performance, the present findings suggest that the actual impact of anxiety-inducing distracters on WM performance is also linked to bottom-up effects. From among the brain regions sensitive to emotional distraction (i.e., showing *in*/*de*creases in activity) (Table 2), only activity in the right visual cortex (BAs 37/18) predicted impaired WM. Similar to the left FG regions showing co-variation with trait anxiety scores, activity in this right visual region was also overall greater in the presence of angry faces. However, it was negatively correlated with WM performance, thus revealing its sensitivity to individual variation in the actual detrimental effect of emotional distraction on performance. This finding provides strong support for the idea that the objective impact of emotional distraction in this task is mainly linked to bottom-up effects, in which enhanced perceptual processing of the anxiety-inducing angry face distracters may divert attention from the main WM task and impair performance.

Taken together, these findings suggest that both the effect of trait anxiety on the general response to transient anxiety-inducing distracters and their actual detrimental impact on WM performance are primarily linked to bottom-up effects involving enhanced activity in perceptual processing brain regions. These findings also suggest a possible dissociation between responses in these regions, reflecting subjective impact and experiencing of anxiety-inducing distraction (left visual cortex and vmPFC) vs. actual/objective impact on WM performance (right visual cortex).

### Patterns of Brain Activity Reflecting the Engagement of Defensive Mechanisms to Cope with Distraction

The present study also identified patterns of correlations that are consistent with a response in brain activity reflecting the engagement of coping mechanisms, as opposed to reflecting impairment by emotional distraction. Specifically, activity in the dorsomedial and left lateral PFC areas was positively correlated with WM performance (i.e., less *de*activation was linked to increased WM performance, see Figure 5), thus providing evidence for a role of these regions in coping with distraction. In other words, participants showing less reduction in the dorsomedial and lateral PFC activity (hence, overall greater activity) also performed better in the WM task, suggesting that they coped better with the presence of task-irrelevant distraction.

These findings are consistent with evidence involving medial and lateral PFC in general cognitive control processing and operations specifically associated with emotion regulation and coping with emotional distraction [16], [17], [56], [57], [58], [59], [60]. Brain imaging studies have implicated the dmPFC, including the dorsal ACC and regions extending dorsally [58] in a variety of cognitive functions, including conflict monitoring [61], [62], complex decision making [63], [64], social interactions [65], and emotion regulation [66], [67]. Similarly, the lateral PFC was linked to processing engaged in coping with emotional distraction [16], [17], [60].

The present dorso-medial and lateral PFC findings in conjunction with the behavioural results suggest that high anxiety aids in performing the WM task with task-irrelevant distraction. The PFC findings showed that less disruptive effects in areas associated with cognitive control and coping with distraction was linked to better WM performance, and behavioral findings showed that high level of anxiety was also associated with better WM performance (see behavioral results). Although the latter finding is in contradiction with evidence that anxiety impairs WM performances [68], it is consistent with the suggestion that people high in anxiety are also more prone to engage compensatory strategies to deal with distraction, in order to maintain standard level of performances [13], [45]. In this context, it is also worth mentioning that these effects are based on activity for correct trials, in which participants were actually able to cope with the distraction, despite the fact that they were overall more affected by the presence of emotional distracters. Also, our participants were not clinically diagnosed with anxiety disorders, and thus it is very likely that the responses in these regions may reflect the engagement of coping strategies probably developed to deal successfully with potentially uncomfortable social situations. In sum, these findings highlight the role of dorso-medial and lateral PFC regions in the actual protection against distraction, by engaging mechanisms of coping with emotional distraction, which may be more effective in participants with higher level of anxiety.

Finally, the present findings also highlight an intriguing hemispheric dissociation and inter-hemispheric relationship between activity in perceptual and executive brain regions and individual variation in trait anxiety and WM performance. Specifically, while individual variations in trait anxiety co-varied with activity in the left perceptual and right executive PFC brain regions, variations in WM performance co-varied with activity in the right perceptual and left executive PFC regions. Although speculative, one possible interpretation is that the inter-hemispheric communication may reflect increased processing engagement to reduce emotional interference [69]. However, further investigations (e.g., using right and left visual field stimulation) [70], along with subjective and objective assessments of the impact of emotional distraction, are needed to clarify these hemispheric dissociations and across-systems inter-hemispheric interactions.

### Caveats

As compelling as it might be, the present investigation also has limitations. One limitation concerns the size of our subject sample, which although allowed identification of robust findings was slightly smaller than the optimal fMRI sample size suggested for investigation of brain-behaviour relationships [71]. Another limitation of the present study is the focus on female participants only, which despite its advantages (as emphasized in Methods) also poses the disadvantage of reduced generalizability of the findings. Thus, it remains to be established whether the present findings identified in healthy female participants can also be generalized to healthy male participants. A third limitation of the present study is the fact that it did not involve comparison of clinical and non-clinical participants. We believe, however, that the involvement of assessments of personality traits indexing individual variation in the targeted emotions (general and specific social anxiety), along with the use of transient anxiety-inducing emotional stimuli as distracters, provide reasonable ecological validity to the present experimental approach. Future studies using similar experimental designs that emphasize the importance of converging evidence from different analytical approaches should further investigate these issues.

### Conclusions

Collectively, the present study provides initial evidence concerning the neural mechanisms sensitive to individual variations in trait anxiety and cognitive performance, which reflects both the detrimental impact of emotion distraction and the engagement of mechanisms to cope with distracting emotions. First, it showed that processing of task-irrelevant anxiety-inducing emotional distracters is associated with opposing patterns of brain activity in affective and executive brain regions, which are modulated by individual variations in both trait anxiety and WM performance. Second, the study also provides evidence that both the effect of trait anxiety on the general response to transient anxiety-inducing distraction and its actual detrimental impact on WM performance are primarily linked to bottom-up effects. Third, the present findings also point to responses engaged to cope with emotional distraction, and highlight the role of specific medial and lateral PFC regions in the actual protection against emotional distraction. These results have implications for understanding alterations in the neural circuitry underlying emotion-cognition interactions in anxiety disorders [72], [73], such as clinical social phobia, in which exacerbated responses to anxiety-inducing social contexts leads to debilitating effects on social behaviour.
